# Nighttime eating during pregnancy and infant adiposity at 6 months of life

**DOI:** 10.3389/fnut.2024.1364722

**Published:** 2024-07-10

**Authors:** Ameyalli M. Rodríguez-Cano, Berenice Medel-Canchola, Isabel González-Ludlow, Carolina Rodríguez-Hernández, Enrique Reyes-Muñoz, Esther Schiffman-Selechnik, Guadalupe Estrada-Gutierrez, Otilia Perichart-Perera

**Affiliations:** ^1^Nutrition and Bioprogramming Coordination, Instituto Nacional de Perinatología Isidro Espinosa de los Reyes, Mexico City, Mexico; ^2^Gynecological and Perinatal Endocrinology Coordination, Instituto Nacional de Perinatología Isidro Espinosa de los Reyes, Mexico City, Mexico; ^3^Private Consultant, Mexico City, Mexico; ^4^Research Division, Instituto Nacional de Perinatología Isidro Espinosa de los Reyes, Mexico City, Mexico

**Keywords:** fat mass, fetal programming, obesity, chrononutrition, prenatal nutrition

## Abstract

**Introduction:**

Chrononutrition studies the relation between diet, circadian rhythms and metabolism, which may alter the metabolic intrauterine environment, influencing infant fat-mass (FM) development and possibly increasing obesity risk.

**Aim:**

To evaluate the association of chrononutrition in pregnancy and infant FM at 6 months.

**Methods:**

Healthy pregnant women and term-babies (*n* = 100pairs) from the OBESO cohort (2017–2023) were studied. Maternal registries included pregestational body-mass-index (BMI), gestational complications/medications, weight gain. Diet (three 24 h-recalls, 1 each trimester) and sleep-schedule (first and third trimesters) were evaluated computing fasting (hours from last–first meal), breakfast and dinner latencies (minutes between wake up-breakfast and dinner-sleep, respectively), number of main meals/day, meal skipping (≥1 main meal/d on three recalls) and nighttime eating (from 9:00 pm–5:59 am on three recalls). Neonatal weight, length, BMI/age were assessed. At 6 months, infant FM (kg, %; air-displacement plethysmography) was measured, and FM index (FMI—kgFM/length^2^) computed. Exclusive breastfeeding (EBF) was recorded. Multiple linear regression models evaluated the association between chrononutrition and 6 month infant FM.

**Results:**

Mean fasting was 11.7 ± 1.3 h; breakfast, dinner latency were 87.3 ± 75.2, 99.6 ± 65.6 min, respectively. Average meals/day were 3.0 ± 0.5. Meal skipping was reported in 3% (*n* = 3) of women and nighttime eating in 35% (*n* = 35). Most neonates had normal BMI/age (88%, *n* = 88). Compared to those who did not, mothers engaged in nighttime-eating had infants with higher %FM (*p* = 0.019). Regression models (*R*^2^ ≥ 0.308, *p* ≤ 0.001) showed that nighttime eating was positively associated with %FM (B: 2.7, 95%CI: 0.32–5.16). When analyzing women without complications/medications (*n* = 80), nighttime eating was associated with higher FM [%FM, B: 3.24 (95%CI: 0.59–5.88); kgFM, B: 0.20 (95%CI: 0.003–0.40); FMI, B: 0.54 (95%CI: 0.03–1.05)]. Infant sex and weight (6 months) were significant, while maternal obesity, pregnancy complications/medications, parity, energy intake, birth-BMI/age, and EBF were not.

**Conclusion:**

Maternal nighttime eating is associated with higher adiposity in 6 month infants.

## 1 Introduction

Infant body composition is a critical determinant of long-term health. Higher adiposity in infancy is associated with an increased risk of metabolic disorders, including obesity, type 2 diabetes, and cardiovascular diseases later in life (1, 2). Different maternal factors (e.g., obesity, gestational weight gain, socioeconomic characteristics) have been identified as influencing infant adiposity (1, 3). Lifestyle and nutrition interventions may play a key role in improving maternal metabolic profiles and reducing the risk of obesity in the next generation (4). Specifically, an optimal diet quality during pregnancy, as well as high adherence to a DASH diet or a Mediterranean dietary pattern has been associated with a better glycemic status and a reduce size and adiposity at birth (5–8).

Dietary assessment has shifted from only measuring the quantity of energy and nutrient intake to more qualitative aspects (type of foods, grade of processing). In addition, the recent study of chrononutrition has documented a relationship between nutrient intake, meal timing patterns, and circadian rhythm with metabolic health (9). Current dietary patterns and meal timing may particularly affect pregnancy, considering it is a period of high metabolic vulnerability due to hormonal and physiological adaptations (10–12). Changes in hormones such as leptin, ghrelin, and insulin, when paired with disrupted circadian rhythms, could lead to impaired hunger signals, increased appetite, and a preference for energy-dense foods (13), contributing to suboptimal dietary patterns and consequently impact fetal growth and adiposity. Evidence about meal timing and chrononutrition behaviors in pregnancy is scarce. Some studies (cross-sectional or observational) have reported an association between nighttime eating, high energy/carbohydrate intake later in the day, and metabolic alterations in pregnant women (14). Generating evidence in this area is imperative as it could facilitate the implementation of simple strategies targeting sleeping and eating schedules for pregnant women.

In non-pregnant adults, disturbances in biological rhythms can alter homeostasis and metabolic processes which have been linked to chronic conditions such as obesity, diabetes, and cardiovascular disease (9, 15). Some chronotypes, particularly the evening type, have been associated with suboptimal glycemic control, higher risk of mortality, and even higher risk of eating disorders (9). The mechanisms explaining the intricate connection between the timing of food consumption throughout the day and regulating the biological clock still need to be clarified.

This study aimed to evaluate if certain chrononutrition behaviors in pregnancy are associated with infant adiposity at 6 months of life.

## 2 Materials and methods

### 2.1 Settings

This study is derived from the institutional perinatal cohort OBESO (Origen Bioquímico y Epigenético del Sobrepeso y la Obesidad), conducted at the Instituto Nacional de Perinatología (Mexico City, Mexico; 2017–2023). The cohort’s main objective was to assess the impact of various maternal characteristics, encompassing clinical, biochemical, lifestyle, and epigenetics aspects, on the neurodevelopment and body composition of the child. The OBESO cohort was conducted by the principles outlined in the Declaration of Helsinki (16). The study was reviewed and approved by the institutional Ethics and Research committees (Project No. 3300-11402-01-575-17). The women’s participation was voluntary, and those who agreed to participate signed the written consent.

### 2.2 Subjects

For this secondary analysis, we included adult pregnant women with singleton pregnancies (recruited between 11.0–13.6 weeks of gestation) with a pregestational body mass index (BMI) ≥18.5, without previous comorbidities (type 2 diabetes, hypertension, uncontrolled thyroid disorders, heart, kidney, liver or autoimmune diseases, or chronic infections HIV, HPV-) or chronic use of medications (affecting carbohydrate or lipid metabolism or markers of inflammation/oxidative stress). Individuals using tobacco, drugs, or alcohol were not included. Exclusion criteria included findings of congenital/structural malformations in the fetus or abnormal fetal karyotype, maternal/fetal infections (chorioamnionitis), and pregnancy loss. We eliminated women without complete follow-up (<3 prenatal visits), those with preterm delivery (<37 weeks, according to first-trimester ultrasound), individuals lacking sleep data, with a nighttime shift or missing three dietary assessments, as well as those with implausible energy intake (<500 or >3,500 kcal/d). Infants without anthropometric or fat mass (FM) measurements at birth or 6 months were eliminated.

### 2.3 Maternal evaluation

Follow-up consisted of three prenatal visits, one in each trimester of pregnancy (first: 11.0 −13.6; second: 18.0 −24.6; third: 28.0 −34.6 weeks of gestation). Initial visit collected socioeconomic data, including age (years), socioeconomic status (very low, low, middle/high), occupation (employee/student, housewife), education (basic: elementary to middle school; middle: high school/technical education; higher: technical career, bachelor’s/graduate degree). The first visit also included clinical information, recording parity (nulliparous: no previous childbirth, multiparous: ≥1 previous childbirth), pregestational weight (self-reported), and height (measured according to Lohman’s technique, (17) to the nearest 0.1 cm, using a digital fixed stadiometer, model 264, SECA, Hamburg, Germany). Pregestational BMI was obtained and classified according to the WHO criteria (18).

During each visit, weight was measured (light clothing, no shoes; measured to the nearest 0.1 kg with a calibrated digital scale, BMB-800, TANITA, Tokyo, Japan), and gestational weight gain (GWG) was calculated based on pregestational weight. GWG was classified according to the Institute of Medicine recommendations (19) as insufficient, adequate, or excessive. In the first and third trimesters, women were queried about their typical nighttime sleep duration (differentiating between hours spent in bed and hours of actual sleep). Women with short sleep were classified [<6 h/night (20)]. Prescribed medications (metformin/insulin), the presence of pregnancy complications (gestational diabetes, preeclampsia), and preterm birth were obtained from the institutional clinical records.

### 2.4 Chrononutrition behaviors and dietary assessment

In each trimester (for a total of 3 evaluations), a trained and experienced nutrition professional conducted dietary assessment using a multiple-pass 24 h recall methodology, having food replicas and standardized measuring cups and spoons to assist in estimating portion sizes. The 24 h recall documented the meal times of the day. The Food Processor SQL software (version 14.0, ESHA Research, Salem, OR, United States) was used to obtain nutrition analysis. The software was previously loaded with standardized local recipes and foods (using food labels or Mexican tables of nutritional value) in its database. Energy (kcal) and grams of protein, carbohydrates, fiber, total fat, monounsaturated, polyunsaturated, saturated fat, and omega-3 fatty acids were registered. The average of the three dietary assessments was computed for each diet component.

The evaluation of chrononutrition behaviors included:

- Fasting (hours): due to the limitation of collecting only one diet recall in each trimester of pregnancy, it was not possible to accurately compute an overnight fasting window between consecutive days. Therefore, we approximated a fasting window by computing the time difference between the final eating occasion and the first eating occasion within a single diet recall, which assumes that the first eating occasion is consistent across days among participants. The average of the approximated fasting hours in the three trimesters was obtained.
- Number of main meals: the count of main meals was determined based on the occasions when food/drinks >250 kcal were consumed in each 24 h recall. The average number of main meals reported across the three visits was also calculated.
- Meal skipping (Yes/No): the classification of meal skipping was established when there were <3 main meals reported in all three of the 24 h recalls.
- Nighttime eating (Yes/No): women who, in all three of the 24 h recalls, reported food consumption outside the daylight period (≥21:00 h and <6:00 h of the next day). The time criteria are established based on local sunset and sunrise times year-round in Mexico.
- Breakfast latency (min): calculated as the time difference in minutes between the moment of waking up (from nighttime sleep duration reported) and the time of the first meal recorded in the 24 h recall. The average of the minutes from the two measurements (first and third trimester) was determined.
- Dinner latency (min): calculated as the time difference in minutes between the last meal consumed in the 24 h recall and bedtime (from nighttime sleep duration reported). The average of the minutes from the two measurements (first and third trimester) was established.

### 2.5 Anthropometric infant assessment

The newborn’s sex, weight, and length (as measured by attending neonatologists) were recorded from institutional records. Gestational age was calculated according to the first-trimester ultrasound. A follow-up visit at 6 months was scheduled for each infant. Following Lohman’s technique, all anthropometric measurements were performed in duplicate (average recorded) by two experienced and trained nutrition professionals (17). Length was measured to the nearest millimeter using an infantometer (SECA 207, Hamburg, Germany). Weight was obtained from the integrated scale on the air-displacement plethysmography device (PEAPOD, COSMED Inc. United States, California, United States). BMI (weight/length2), was computed and BMI for age (BMI/age) *z*-score and classification were obtained based on the WHO reference (21).

### 2.6 Fat mass measurement

At 6 months, infant FM estimation was performed using the PEAPOD device (COSMED Inc. United States, California, United States). Prior to measurement, the PEAPOD was calibrated (following the protocol in the manufacturer’s manual). The device was then configured with the child’s data, including the day of birth, gestational age, sex, and length. To prepare the infant for measurement, they were undressed, and a cap was placed on their head. The FM measurement commenced with weight measurement by placing the infant on the integrated scale of the PEAPOD. Following this, the infant was positioned inside the PEAPOD chamber for body volume measurement, and subsequently, body density was calculated. Finally, the PEAPOD’s software employed Fomon’s equation (22) to compute the infant’s FM (in kg—kgFM—and as a percentage—%FM). Fat mass index (FMI) was calculated by dividing kgFM by length^2^ (m) (23, 24).

### 2.7 Breastfeeding practices

At 6 months, the classification of exclusive breastfeeding (EBF) was determined through interviews with mothers regarding the feeding practices of their infants during the first 6 months. EBF included the consumption of breast milk, water, non-sweetened herbal teas, oral rehydration solutions, drops, and syrups. Non-EBF infants received any amount of formula, whether in combination with breastmilk or as the sole source of feeding.

### 2.8 Statistical analysis

Descriptive statistics were employed to present the population’s characteristics (socioeconomic, clinical data) and the description of the maternal diet, chrononutrition habits, and infant FM. Bivariate analyses were performed: correlations of continuous variables (Pearson/Spearman); mean differences between groups (Student’s *t*-test/U-Mann Whitney test, one-way ANOVA/Kruskal Wallis test); and the difference between two proportions (Chi-square test). These analyses aimed to explore chrononutrition behaviors and infant FM according to maternal socioeconomic and clinical characteristics, energy, and nutrient consumption according to chrononutrition behaviors, and to assess the association between the different chrononutrition habits (independently) and infant FM.

To study the influence of individual chrononutrition behaviors (fasting hours, number of main meals, meal skipping, nighttime eating, breakfast latency, and dinner latency) on infant FM (kgFM, %FM, FMI), we performed multiple linear regression models. Each model incorporated confounding variables for adjustment, selected based on (1) their recognized influence on fat mass according to existing literature and (2) previous bivariate analyses. These confounding variables encompassed maternal pregestational BMI, parity, pregnancy complications, metformin/insulin use, energy intake during pregnancy, infant sex, birth BMI/age, and EBF. The strength of the models was assessed through *R*^2^ values. Normal distribution of residuals was verified to ensure the assumptions of the regression models. Sensitivity analyses were conducted, considering other potential maternal influences on infant FM (e.g., total fat intake, saturated fat, monounsaturated fat, and sleep hours/short sleep). Variables without significant impact on the models (no change in *R*^2^ or *p*-value) were excluded, and only the final models are presented. A *p*-value <0.05 was considered statistically significant. All statistical analyses were performed using the SPSS software version 26.0 (IBM, Armonk, NY, United States).

## 3 Results

Out of the 502 women enrolled in the OBESO cohort, 44 were excluded due to the presence of chronic or uncontrolled diseases (20 with type 2 diabetes, 13 with hypertension, and 11 with uncontrolled hypothyroidism). Additionally, 183 women were eliminated as they did not attend at least three prenatal visits. Eighty-six women had incomplete dietary information, and one of them was excluded because of a nighttime shift. No woman exhibited an implausible energy intake (<500 or >3,500 kcal/d).

Furthermore, 88 infants were eliminated from the analysis because they lacked FM measurement (non-attendance at visit, age below 6 months, not measured for FM) or birthweight. A total of 100 mother-infant pairs were included in the final analysis.

### 3.1 Description of pregnant women and chrononutrition behaviors during pregnancy

Table 1 shows the maternal characteristics of our population. The majority of women were nulliparous, were housewives, had middle to higher level of education, and had very low/low socioeconomic status. Most women presented overweight/obesity, and the majority presented excessive GWG. Pregnancy complications and the use of metformin/insulin were observed in only a small percentage of the sample. Sleep had a minimum duration of 1.64 h and a maximum of 10 h. More than a third of women presented short sleep.

**Table 1:** Maternal characteristics according to nighttime eating during pregnancy.

Maternal characteristics	All (*n* = 100)	Nighttime eating		*p*-value
		No (*n* = 65)	Yes (*n* = 35)	
Age (years)	30.7 ± 4.9	30.5 ± 4.9	31.1 ± 4.9	0.401
Pregestational BMI (kg/m^2^)	27.3 ± 5.3	27.2 ± 5.0	27.4 ± 6.0	0.971
Pregestational BMI status				
Normal, *n* (%)	41 (41.0)	27 (65.9)	14 (34.1)	0.979
Overweight, *n* (%)	33 (33.0)	21 (63.6)	12 (36.4)	
Obesity, *n* (%)	26 (26.0)	17 (65.4)	9 (34.6)	
Parity				
Nulliparous, *n* (%)	65 (65.0)	40 (61.5)	25 (38.5)	0.383
Multiparous, *n* (%)	35 (35.0)	25 (71.4)	10 (28.6)	
Gestational weight gain				
Insufficient, *n* (%)	32 (32.0)	20 (62.5)	12 (37.5)	0.840
Adequate, *n* (%)	24 (24.0)	15 (62.5)	9 (37.5)	
Excessive, *n* (%)	44 (44.0)	30 (68.2)	14 (31.8)	
Pregnancy complications				
Yes, *n* (%)	13 (13.0)	11 (84.6)	2 (15.4)	0.132
No, *n* (%)	87 (87.0)	54 (62.1)	33 (37.9)	
Metformin/Insulin use				
Yes, *n* (%)	10 (10.0)	8 (80.0)	2 (20.0)	0.487
No, *n* (%)	90 (90.0)	57 (63.3)	33 (36.7)	
Education				
Basic, *n* (%)	18 (18.0)	11 (61.1)	97 (38.9)	0.221
Middle, *n* (%)	40 (40.0)	30 (75.0)	10 (25.0)	
Higher, *n* (%)	42 (42.0)	24 (57.1)	18 (42.9)	
Socioeconomic status				
Very low, *n* (%)	10 (10.0)	5 (50.0)	5 (50.0)	0.571
Low, *n* (%)	44 (44.0)	29 (65.9)	15 (34.1)	
Middle/high, *n* (%)	46 (46.0)	31 (67.4)	15 (32.6)	
Occupation				
Housewife, *n* (%)	60 (63.8)	44 (73.3)	16 (26.7)	0.111
Employee/student, *n* (%)	34 (36.2)	19 (55.9)	15 (44.1)	
Sleep				
Mean sleep (hr)	6.3 ± 1.7	6.3 ± 1.6	6.1 ± 1.8	0.489
Short sleep (<6 h)				
Yes, *n* (%)	38 (38.4)	24 (63.2)	14 (36.8)	0.832
No, *n* (%)	61 (61.6)	40 (65.6)	21 (34.4)	

Data presented as mean ± standard deviation or as number of cases and percentage in parenthesis. Test performed: student‘s *t* test/U Mann–Whitney test or Chi-squared test.

Most women had three main meals per day (2.0–5.67 meals); only 3% (*n* = 3) of them reported meal skipping during pregnancy. Approximately one-third of women (35%, *n* = 35) presented nighttime eating during gestation. The mean breakfast and dinner latency duration were 87.3 ± 75.2 min and 99.6 ± 65.6, respectively, with a wide distribution (breakfast range: 0 to 320 min, dinner: 0 to 330 min). The mean night fasting duration was 11.7 ± 1.3 h (6.52 to 14.75 h).

Nulliparous women showed a shorter duration of breakfast latency (min, 71.38 ± 64.84 vs. 117.07 ± 84.67, *p* = 0.009) and longer sleep hours (hr, 6.6 ± 1.6 vs. 5.7 ± 1.7, *p* = 0.012). No other differences in chrononutrition behaviors were found based on clinical (pregestational BMI, GWG) or maternal socioeconomic variables (occupation, socioeconomic status, education). Maternal age exhibited a positive correlation with the number of main meals per day (*r* = 0.201, *p* = 0.045) and a negative correlation with hours of fasting (*r* = −0.292, *p* = 0.003) and sleeping (*r* = −0.286, *p* = 0.004). No additional correlations were observed between pregestational BMI and chrononutrition behaviors.

Table 2 describes the mean consumption of energy and macronutrients during pregnancy. Women with pregestational obesity presented a lower consumption of carbohydrates (grams, Normal: 286.8 ± 69.8, Overweight: 260.2 ± 58.7, Obesity: 246.9 ± 57.0, *p* = 0.034) and saturated fat (grams, Normal: 22.5 ± 9.2, Overweight: 20.3 ± 7.1, Obesity: 18.2 ± 8.0, *p* = 0.041). No energy or nutrient consumption differences were identified according to GWG, parity, occupation, socioeconomic status, education, or short sleep. Sleep hours did not correlate with energy or nutrient intake.

**Table 2:** Energy and nutrient consumption during pregnancy according to nighttime eating.

Nutrient	All (*n* = 100)	Nighttime eating		*p*-value
		No (*n* = 65)	Yes (*n* = 35)	
Energy (kcal)	2057.9 ± 498.2	2011.2 ± 517.1	2144.7 ± 455.6	0.203
Protein (g)	86.7 ± 23.0	85.2 ± 24.4	89.4 ± 20.1	0.388
Carbohydrates (g)	267.8 ± 64.7	266.3 ± 65.2	270.6 ± 64.7	0.757
Fiber (g)	26.2 ± 9.3	26.5 ± 10.1	25.5 ± 7.7	0.618
Fat (g)	73.2 ± 24.2	69.6 ± 24.7	79.9 ± 21.9	0.023
Saturated fat (g)	20.7 ± 8.4	19.6 ± 8.6	22.8 ± 7.6	0.025
Monounsaturated fat (g)	23.1 ± 8.1	21.8 ± 8.1	25.5 ± 7.7	0.029
Polyunsaturated fat (g)	14.1 ± 6.1	13.8 ± 6.3	14.5 ± 5.8	0.515
Omega-3 (g)	1.2 ± 0.6	1.2 ± 0.7	1.2 ± 0.5	0.997

Data presented as mean ± standard deviation. Test performed: student’s t test/U Mann–Whitney test.

Some consumption differences were identified based on chrononutrition behaviors. In women with meal skipping, there was a significantly higher intake of protein (grams, 87.6 ± 22.4 vs. 56.3 ± 24.2, *p* = 0.033) and a trend of higher energy (kcals, 2073.4 ± 494.4 vs. 1558.9 ± 414.1, *p* = 0.078) and total fat intake (grams, 74.0 ± 23.9 vs. 46.8 ± 23.1, *p* = 0.067).

The number of main meals per day demonstrated a positive correlation with protein (*r* = 0.330, *p* = 0.001) and fiber (*r* = 0.344, *p* < 0.001) consumption, while breakfast latency showed a negative correlation with omega-3 fatty acids intake (*r* = −0.214, *p* = 0.032). Fasting hours were negatively correlated with energy (*r* = −0.222, *p* = 0.027), protein (*r* = −0.250, *p* = 0.012), fiber (*r* = −0.259, *p* = 0.009), total fat (*r* = −0.250, *p* = 0.012), monounsaturated (*r* = −0.343, *p* < 0.001) and polyunsaturated (*r* = −0.307, *p* = 0.002) fat and omega-3 fatty acids intake (*r* = −0.266, *p* = 0.007). Women with nighttime eating presented higher consumption of total fat (*p* = 0.023), saturated (*p* = 0.025) and monounsaturated fat (*p* = 0.029) (Table 2).

### 3.2 Description of newborn and infant anthropometry and infant FM

The mean gestational age of newborns was 38.9 ± 1.0 weeks, with 52% (*n* = 52) of female infants. Anthropometric measurements at birth and 6 months and adiposity indicators are detailed in Table 3. Most infants were classified with normal BMI/age at birth, and 8% (*n* = 8) and 3% (*n* = 3) presented overweight risk and overweight, respectively; no case of obesity was detected. No differences in anthropometric measurements or BMI/age classification were observed at birth based on sex. At 6 months, 12% (*n* = 12) of infants were at risk of overweight and 2% (*n* = 2) were classified as overweight; there were no cases of obesity detected. Boys presented higher weight (kg, 7.0 ± 0.8 vs. 7.6 ± 0.8, *p* = 0.001), length (cm, 64.4 ± 2.3 vs. 65.8 ± 2.0, *p* = 0.002), and BMI (kg/m^2^, 16.9 ± 1.3 vs. 17.5 ± 1.5, *p* = 0.032) compared to girls, but no difference in BMI/age classification at 6 months. No differences in adiposity (%FM, kgFM, FMI) were found between boys and girls. Thirty percent (*n* = 30) of infants received EBF for the first 6 months of life. According to EBF, there were no differences in anthropometric or adiposity measurements at 6 months. According to maternal clinical, socioeconomic, and sleep categories, there were no differences in infant anthropometric measurements or BMI/age classification at 6 months.

**Table 3:** Description of newborn and infant anthropometric measurements and infant FM according to maternal nighttime eating.

Anthropometric/FM measurements	All (*n* = 100)	Nighttime eating		*p*-value
		No (*n* = 65)	Yes (*n* = 35)	
Sex				
Female, *n* (%)	52 (52.0)	33 (63.5)	19 (36.5)	0.835
Male, *n* (%)	48 (48.0)	32 (66.7)	16 (33.3)	
BIRTH				
Weight (kg)	2.9 ± 0.3	2.9 ± 0.3	2.9 ± 0.4	0.480
Length (cm)	47.1 ± 1.7	47.1 ± 1.6	47.0 ± 1.9	0.942
BMI (*z*-score)	−0.19 ± 0.98	−0.26 ± 0.93	−0.06 ± 1.05	0.330
BMI/age category				
Wasting, *n* (%)	1 (1.0)	1(100.0)	0 (0.0)	0.714
Normal, *n* (%)	88 (88.0)	58 (65.9)	30 (34.1)	
Overweight risk, *n* (%)	8 (8.0)	4 (50.0)	4 (50.0)	
Overweight, *n* (%)	3 (3.0)	2 (66.7)	1 (33.3)	
SIX MONTHS				
Weight (kg)	7.3 ± 0.8	7.2 ± 0.9	7.3 ± 0.8	0.598
Length (cm)	65.1 ± 2.3	65.1 ± 2.3	65.1 ± 2.3	0.914
BMI (*z*-score)	0.01 ± 0.97	−0.04 ± 0.99	0.12 ± 0.93	0.401
BMI/age category				
Wasting, *n* (%)	3 (3.0)	2 (66.7)	1 (33.3)	0.783
Normal, *n* (%)	83 (83.0)	55 (66.3)	28 (33.7)	
Overweight risk, *n* (%)	12 (12.0)	7 (58.3)	5 (41.7)	
Overweight, *n* (%)	2 (2.0)	1 (50.0)	1 (50.0)	
Adiposity				
FM (%)	23.7 ± 6.3	22.6 ± 6.3	25.7 ± 5.9	0.019
FM (kg)	1.77 ± 0.59	1.69 ± 0.58	1.92 ± 0.60	0.067
FMI (kgFM/length^2^)	4.15 ± 1.32	3.96 ± 1.27	4.51 ± 1.35	0.046
Exclusive breastfeeding for 6 months				
Yes, *n* (%)	30 (30.3)	19 (63.3)	11 (36.7)	0.857
No, *n* (%)	70 (70.0)	46 (65.7)	24 (34.3)	

Data presented as mean ± standard deviation or as number of cases and percentage in parenthesis. Test performed: student’s *t* test/U Mann–Whitney test or Chi-squared test. FM: fat mass; BMI: body mass index; FMI: fat mass index.

Regarding adiposity, infants born to mothers with pregestational obesity exhibited lower FM (*p* < 0.05). No other differences in adiposity indicators according to GWG, parity, sleep, or any maternal socioeconomic characteristics were found.

### 3.3 Chrononutrition behaviors and their association with infant FM at 6 months

Infants born to nighttime-eating mothers had higher %FM (*p* = 0.019) and FMI (*p* = 0.046) and a tendency towards higher kgFM (*p* = 0.067) (Table 3). No differences in anthropometry or adiposity were identified based on main meal skipping. A negative correlation between breakfast latency and infant weight (*r* = −0.258, *p* = 0.009) and length (*r* = −0.317, *p* = 0.001) was observed at 6 months. No other differences were found between infant anthropometric or adipositymeasurements and chrononutrition behaviors.

In the multiple linear regression models, the only significant model (*R*^2^: 0.311, *p* < 0.001) was for maternal nighttime eating, which was associated with a significantly higher %FM and a trend towards higher kgFM and FMI (Table 4). Infant sex and weight at 6 months were also found to be associated factors. These associations were independent of maternal pregestational BMI, parity, pregnancy complications, metformin/insulin use, total energy intake, birth BMI/age, and EBF for 6 months. The total fat, saturated fat, monounsaturated fat intake, and sleep did not modify the models.

**Table 4:** Association of nighttime eating during pregnancy and infant fat at 6 months of life, including all women in the sample.

Predictive variables	KG FM		% FM		FMI	
	*R*^2^: 0.555, *p* < 0.001		*R*^2^: 0.311, *p* < 0.001		*R*^2^: 0.431, *p* < 0.001	
	B (95%CI)	*p*	B (95%CI)	*p*	B (95%CI)	*p*
Constant	−1.42 (−2.43 to −0.41)	0.006	5.58 (−9.78 to 16.96)	0.595	−1.29 (−3.83 to 1.23)	0.312
Pregestational BMI (kg/m^2^)	−0.01 (−0.02 to 0.006)	0.228	−0.10 (−0.32 to 0.11)	0.324	−0.02 (−0.06 to 0.01)	0.249
Parity: Multiparous	−0.03 (−0.22 to 0.34)	0.682	−0.05 (−2.50 to 2.39)	0.965	−0.04 (−0.50 to 0.42)	0.856
Pregnancy complications: Yes	0.04 (−0.22 to 0.31)	0.742	0.64 (−2.94 to 4.25)	0.723	0.10 (−0.58 to 0.78)	0.762
Metformin/insulin use: Yes	−0.13 (−0.44 to 0.17)	0.386	−1.91 (−5.98 to 2.14)	0.351	−0.37 (−1.14 to 0.39)	0.340
Energy intake (kcal)	−2.7 E-05 (0.00 to 0.00)	0.761	−0.001 (−0.003 to 0.002)	0.592	0.00 (−0.001 to 0.00)	0.470
Nighttime eating: Yes	0.17 (−0.012 to 0.354)	0.066	2.74 (0.32 to 5.16)	0.027	0.43 (−0.02 to 0.89)	0.061
Sex: Male	−0.25 (−0.43 to −0.06)	0.009	−3.15 (−5.61 to −0.70)	0.012	−0.62 (−1.09 to −0.16)	0.009
Newborn BMI/age (*z*-score)	0.01 (−0.08 to 0.10)	0.785	0.23 (−1.01 to 1.48)	0.709	0.09 (−0.14 to 0.33)	0.424
Weight 6 months (kg)	0.49 (0.38 to 0.59)	<0.001	3.39 (1.94 to 4.83)	<0.001	0.89 (0.62 to 1.17)	<0.001
EBF for 6 months: Yes	0.08 (−0.11 to 0.27)	0.417	0.92 (−1.66 to 3.50)	0.482	0.25 (−0.23 to 0.74)	0.309

Multiple linear regression models. FM, fat mass; FMI, fat mass index; BMI, body mass index; EBF, exclusive breastfeeding.

After excluding women with pregnancy complications and medication use (*n* = 20), multiple linear regression models showed that having a mother engaged in nighttime consumption during pregnancy was associated with a higher %FM, kgFM, and FMI at 6 months, as well as being female or having a higher infant body weight (Table 5). These associations were independent of maternal pregestational BMI, parity, total energy intake, birth BMI/age, and EBF for 6 months. Intake of total fat, saturated and monounsaturated fat did not modify the models. None of the models performed for the other chrononutrition behaviors were significant for this subgroup of women.

**Table 5:** Association of nighttime eating during pregnancy in women without pregnancy complications or medication use and infant fat at 6 months of life.

Predictive variables	KG FM		% FM		FMI	
	*R*^2^: 0.544, *p* < 0.001		*R*^2^: 0.308, *p* = 0.001		*R*^2^: 0.427, *p* = 0.001	
	B (95%CI)	*p*	B (95%CI)	*p*	B (95%CI)	*p*
Constant	−1.26 (−2.38 to −0.14)	0.028	5.87 (−8.88 to 20.62)	0.430	−0.972 (−3.82 to 1.88)	0.500
Pregestational BMI (kg/m^2^)	−0.012 (−0.02 to 0.006)	0.188	−0.12 (−0.35 to 0.10)	0.268	−0.028 (−0.72 to 0.01)	0.211
Parity: Multiparous	−0.003 (−0.20 to 0.20)	0.973	0.473 (−2.23 to 3.18)	0.729	0.036 (−0.48 to 0.01)	0.891
Energy intake (kcal)	−3.66 E-05 (−0.00 to 0.00)	0.742	−0.001 (−0.004 to 0.002)	0.539	0.00 (−0.001 to 0.00)	0.349
Nighttime eating: Yes	0.20 (0.003 to 0.40)	0.046	3.24 (0.59 to 5.88)	0.017	0.54 (0.03 to 1.05)	0.036
Sex: Male	−0.29 (−0.50 to −0.08)	0.007	−3.68 (−6.42 to −0.93)	0.009	−0.75 (−1.28 to −0.22)	0.006
Newborn BMI/age (*z*-score)	0.008 (−0.09 to 0.11)	0.887	0.12 (−1.27 to 1.51)	0.863	0.64 (−0.20 to 0.33)	0.640
Weight 6 months (kg)	0.47 (0.35 to 0.60)	<0.001	3.22 (1.57 to 4.87)	<0.001	0.88 (0.58 to 1.21)	<0.001
EBF for 6 months: Yes	0.01 (−0.19 to 0.22)	0.896	−0.07 (−2.83 to 2.68)	0.956	0.078 (−0.45 to 0.61)	0.772

Multiple linear regression models. FM, fat mass; FMI, fat mass index; BMI, body mass index; EBF, exclusive breastfeeding.

## 4 Discussion

This is one of the first prospective studies that describe chrononutrition behaviors during pregnancy (meal skipping, morning latency, and nighttime eating) and shows some influence on maternal nutrient consumption (protein, total fat, saturated and monounsaturated fat, omega-3 fatty acids) and infant adiposity. Our findings suggest a potential link between maternal nighttime eating during pregnancy and higher infant adiposity at 6 months.

Around 30% of our population engaged in nighttime eating, a prevalence that falls within the range of 15–45% documented in other reports on pregnant women (12, 25, 26). In our study, maternal nighttime eating was associated with a higher infant adiposity. This could be related to circadian rhythm disruption by nighttime eating. Glucose sensitivity typically peaks during daylight, as insulin release responds to circadian cycles, with pancreatic β-cells having maximum activity around midday. This suggests that food intake might be more efficiently metabolized during the day, as postprandial glycemic digressions are more significant in the evening (15, 27). The liver, crucial in nutrient metabolism, is strongly connected to feeding patterns and can alter the phase of liver clock genes. Consuming food out of daylight may increase plasmatic non-esterified fatty acids and reduce insulin sensitivity (15, 27). Among chronotypes, the evening type is associated with suboptimal glycemic control (9); moreover, people with diabetes with night eating syndrome are more susceptible to obesity, suboptimal HbA1c values, and diabetic complications (28). In the context of pregnancy, nighttime eating was associated with higher maternal glucose values (25, 29) and inversely associated with early-phase insulin secretion (29) in two different cohorts of Asian and African American pregnant women. Turkish pregnant women classified with night eating syndrome also presented greater HOMA-IR (Homeostasis model assessment of insulin resistance), insulin, total cholesterol, and high-density lipoprotein cholesterol (30). Maternal hyperglycemia during pregnancy predisposes the offspring to a higher risk of obesity or higher FM at birth and in childhood (31, 32). A recent systematic review observed that interventions to improve glycaemia during gestation showed a protective effect on infant adiposity (33). While glucose regulation is one of the metabolic processes most studied about chrononutrition, many other mechanisms may be involved. During pregnancy, it has been documented that some characteristics of chrononutrition could affect the secretion of melatonin and cortisol (12), which influence different metabolic processes such as oxidative stress, inflammation, the renin–angiotensin system, epigenetic regulation, among others (11). Higher cortisol concentrations and melatonin concentrations have been associated with adverse perinatal outcomes (34–36). Gestational cortisol is also linked to infant adiposity during the first 6 months of life (37). There is an intricate interplay between circadian rhythm, nutrition, and metabolic processes during pregnancy, underlying the need for further research in this complex area. Manipulating meal timing may be a potential strategy to manage glucose homeostasis, improve metabolic health, and prevent adverse perinatal outcomes.

Nighttime eating was also related to higher consumption of fat (total, saturated, monounsaturated). Previous studies in non-pregnant population have associated evening-eating patterns with higher consumption of unhealthy foods (ultraprocessed products, sweets, sweetened beverages, high-fat foods, fast foods, alcohol) and lower intake of healthy food groups (fruits, vegetables, fish) (38). In other studies, in pregnant women, nighttime eating was associated with higher fat and lower calcium, iron, and riboflavin intake (25, 26). While a low-quality dietary pattern, a high-fat diet, and higher saturated fat consumption during pregnancy have been related to higher neonatal adiposity (39–42), our models did not reveal an effect of total or specific fat intake on infant FM. Maternal nutrition is one of the main determinants of maternal-fetal well-being (43–45). Nighttime eating in pregnancy has been associated with shorter gestation length (−0.45 weeks 95%CI: −0.75 to −0.16) and a higher risk of preterm birth (OR 2.19, 95%CI: 1.01 to 4.72) in Asian women (46).

Most women in our study reported having three main meals per day, and almost no one skipped main meals during gestation. Other studies have reported a high percentage of pregnant women having at least three meals per day women [95.6% of a sample of Polish women (47) and 75.6% of a U.S.-based cohort (48)]. Meal frequency could represent an essential aspect of prenatal nutrition, as recommended by guidelines advocating three meals (in addition to snacks) to adequately address the nutritional demands of pregnancy (49), to reduce the risk of birth defects, inadequate fetal growth and development, and adverse chronic disorders for the mother-infant binomial (50). Our results showed an association of higher protein intake (and a trend of higher energy and fat intake) in those women not skipping meals. Other authors have revealed a lower protein intake, lower plasma docosahexaenoic and eicosapentaenoic acid and beta-carotene (51), iron deficiency (52), and even undernutrition (low mid-upper arm circumference) in pregnant women who skip meals (52). Furthermore, skipping meals may alter various physiological mechanisms due to lower dietary consumption and different factors related to chrononutrition. Skipping meals could trigger a stress response, elevating prostaglandins, adrenaline, and insulin levels, potentially contributing to adverse perinatal outcomes (48, 53). The study performed by Siega-Riz and cols. Showed that women with fewer meals/day had a relative risk of 1.57 (95%CI: 0.88 to 2.79) of early preterm and 1.22 (95%CI: 0.86 to 1.73) of late preterm delivery, even after adjusting for confounding variables such as energy intake (pregestational BMI and supplement use) (48). A more recent study by Englund-Ögge et al. also found that routine consumption of three main meals was related to a lower preterm risk (hazard ratio: 0.89, 95%CI: 0.80 to 0.98, *p* = 0.028) (adjusted by fiber as an indicator of a healthy diet) (53). Regular breakfast skipping during pregnancy has also been associated with a higher risk of gestational diabetes (54).

More extended night fasting has been related to lower energy intake (55) in accordance with the negative correlation coefficients between fasting hours and general intake we observed. The length of overnight fasting has also been negatively associated with fasting glucose, independent of energy and macronutrient intake, which could reflect clock-related mechanisms of metabolism regulation (55). Women in our sample reported a fasting period close to 12 h, although it is only an approximation and not a true overnight fasting window. Other authors have reported 9.9 h in an Asian perinatal cohort (55) and 13 h in Brazilian pregnancies (38), reflecting the influence of sociocultural factors on diet. Given the current scarcity of research, there is uncertainty regarding what constitutes a healthy duration of fasting time and the potential effects of usual fasting periods on perinatal outcomes. A broader set of studies have assessed Ramadan fasting and perinatal outcomes, but this type of practice differs from a non-religious habitual dietary behavior. A recent meta-analysis showed that Ramadan-related fasting affected lower GWG during fasting but did not affect weight gain throughout pregnancy, gestational age at birth, preterm birth, birthweight, low birthweight, or other maternal-infant outcomes (56).

Breakfast and dinner latencies represent novel elements scarcely studied within the chrononutrition field. These aspects could be associated with alterations in consumption and metabolic issues related to light/dark periods. Our results show that the shorter time between waking up and having breakfast was associated with higher consumption of omega-3 fatty acids, which may be a random finding. Exploring these timing factors and their association with maternal-infant health is essential.

Despite the potential influence of sleep on various aspects of the diet, we found no associations between sleep duration and energy/nutrient consumption or any of the chrononutrition behaviors evaluated. A study in healthy pregnant women by Van Lee and cols. Found no association between sleep quality or sleep hours and fasting, number of meals, or nighttime eating. The only identified association was that good quality sleep was associated with fewer discretionary calories (beverages, cakes, desserts, snacks) (57).

EBF is widely considered the standard in infant nutrition for the first 6 months of life. While in this specific analysis, EBF was not associated with FM, other studies conducted in this early stage show that EBF is an influential factor contributing to differences in FM (58, 59). Given its substantial impact on infant health, including benefits such as reduced mortality, lower rates of infections, positive effects on neurodevelopment (60), and the protective effect against adult obesity (61), promoting breastfeeding should be a priority.

Maternal obesity is recognized as a factor that predisposes the offspring to alterations in fetal growth (62), with excessive and insufficient growth associated with higher adiposity later in life (63). In our bivariate analysis, we found a lower FM in infants born to mothers with gestational obesity. However, pregestational BMI was not associated with FM in our multiple regression models examining the association between maternal nighttime eating and adiposity.

This study presents some limitations that warrant consideration. Firstly, the absence of a specific dietary assessment method to evaluate chrononutrition behaviors prospectively may have introduced error and different biases in the estimation of our indicators. The fact that we analyzed information from each recall and used the average for computing chrononutrition variables is an indirect assessment, especially regarding the approximated overnight fasting duration used in our analysis. In addition, the study design did not allow us to assess or consider differences in chrononutrition behaviors across pregnancy trimesters. It is possible that the behaviors described in this study are not representative of all stages of pregnancy. Although we were able to assess usual sleeping schedule, the retrospective nature of the questionnaire is not ideal, and may have also introduced error.

Additionally, a more comprehensive evaluation of episodes of night eating could have included the proportion of calories or a macronutrient analysis, providing a more detailed understanding of dietary patterns. It is also possible that women under or overreported energy/macronutrient consumption, potentially confounding some results. The lack of FM measurement at birth is another limitation, as having this variable could have allowed for better adjustment in our models or facilitated a longitudinal analysis. On the other hand, considering the sample size of this study, the estimated power is 73%, which is a reasonable value. However, it may somehow decrease the reliability and precision of our findings. The results of this study may not be applicable for the general population, considering ethnicity, socioeconomic status and that women receiving prenatal care in our hospital are classified as “high-risk” pregnancies. Even though we established strict inclusion criteria in this cohort for reducing variability, the external validity may be low.

This study possesses several strengths. Using data from a prospective cohort enabled the assessment of maternal variables throughout pregnancy and early infancy. This longitudinal approach allowed for considering various influential factors on adiposity in our statistical models. The ability to evaluate dietary consumption longitudinally, and with at least three recalls, allowed a better characterization of chrononutrition behaviors throughout pregnancy. Air-displacement plethysmography is a well-validated method to assess FM in this early stage of life. Our outcome variable included different indicators of adiposity such as a percentage (relative), kilograms (absolute), and FMI (relative to length) to have a broader analysis with better indicators of body composition.

The temporal aspects of feeding should be included when evaluating dietary patterns in pregnant women. Different aspects of chrononutrition, including meal skipping, breakfast latency, and nighttime eating, could affect the intake of nutrients and may indicate different nutrition and metabolic imbalances.

## 5 Conclusion

Maternal nighttime eating is associated with higher infant adiposity at 6 months of age, independently of maternal obesity, use of metformin or insulin, energy intake in pregnancy, and exclusive breastfeeding. Chrononutrition behavior modification could represent an innovative and feasible strategy that may be incorporated into nutritional counseling during pregnancy for improving nutrient intake and promoting metabolic health.

## Data availability statement

The raw data supporting the conclusions of this article will be made available by the authors, without undue reservation.

## Ethics statement

The studies involving humans were approved by Ethics and Research committees—Instituto Nacional de Perinatología (Project No. 3300-11402-01-575-17). The studies were conducted in accordance with the local legislation and institutional requirements. The participants provided their written informed consent to participate in this study.

## Author contributions

AR-C: Conceptualization, Data curation, Formal analysis, Visualization, Writing – original draft. BM-C: Data curation, Writing – review & editing. IG-L: Investigation, Writing – review & editing. CR-H: Investigation, Writing – review & editing. ER-M: Conceptualization, Project administration, Writing – review & editing. ES-S: Data curation, Writing – review & editing. GE-G: Conceptualization, Funding acquisition, Project administration, Writing – review & editing. OP-P: Conceptualization, Methodology, Project administration, Supervision, Writing – review & editing.
